# Respiratory motion-corrected T1 mapping of the abdomen

**DOI:** 10.1007/s10334-024-01196-1

**Published:** 2024-08-12

**Authors:** Jana Huiyue Zhang, Tom Neumann, Tobias Schaeffter, Christoph Kolbitsch, Kirsten Miriam Kerkering

**Affiliations:** 1 Physikalisch-Technische Bundesanstalt (PTB), Braunschweig and Berlin, Germany; 2 Department of Biomedical Engineering, Technical University of Berlin, Berlin, Germany; 3 Department of Diagnostic and Interventional Radiology, Lausanne University Hospital (CHUV) and University of Lausanne (UNIL), Lausanne, Switzerland; 4 School of Biomedical Engineering and Imaging Sciences, King’s College London, London, United Kingdom

**Keywords:** Abdominal T1 mapping, Respiratory motion correction, Image-based navigator

## Abstract

**Objective:**

The purpose of this study was to investigate an approach for motion-corrected T1 mapping of the abdomen that allows for free breathing data acquisition with 100% scan efficiency.

**Materials and methods:**

Data were acquired using a continuous golden radial trajectory and multiple inversion pulses. For the correction of respiratory motion, motion estimation based on a surrogate was performed from the same data used for T1 mapping. Image-based self-navigation allowed for binning and reconstruction of respiratory-resolved images, which were used for the estimation of respiratory motion fields. Finally, motion-corrected T1 maps were calculated from the data applying the estimated motion fields. The method was evaluated in five healthy volunteers. For the assessment of the image-based navigator, we compared it to a simultaneously acquired ultrawide band radar signal. Motion-corrected T1 maps were evaluated qualitatively and quantitatively for different scan times.

**Results:**

For all volunteers, the motion-corrected T1 maps showed fewer motion artifacts in the liver as well as sharper kidney structures and blood vessels compared to uncorrected T1 maps. Moreover, the relative error to the reference breathhold T1 maps could be reduced from up to 25% for the uncorrected T1 maps to below 10% for the motion-corrected maps for the average value of a region of interest, while the scan time could be reduced to 6-8 s.

**Discussion:**

The proposed approach allows for respiratory motion-corrected T1 mapping in the abdomen and ensures accurate T1 maps without the need for any breathholds.

**Supplementary Information:**

The online version contains supplementary material available at 10.1007/s10334-024-01196-1.

## Introduction

Quantitative T1 mapping of the abdomen serves as a non-invasive imaging technique that can help to characterize pathologies and monitor therapy effects in organs of the abdominal region. For example, T1 mapping can be employed to distinguish between a healthy and a fatty liver [1], for assessing the different fibrotic states of the liver [2] or to detect liver cirrhosis [3]. It is further possible to use T1 mapping for the evaluation of the kidney function [4, 5] or to diagnose mild chronic pancreatitis in the pancreas [6]. The first step for obtaining a T1 map is to acquire a set of T1-weighted images, where each image is acquired at a different time point after the application of a magnetization preparation radio-frequency (RF) pulse. In a second step, the images are fitted to a signal model describing the behavior of the longitudinal magnetization over time. One approach to obtain T1 maps is to acquire a single readout after the application of the preparation pulse and then to wait till the longitudinal magnetization has completely recovered. After full recovery, the preparation RF-pulse is applied again and the next readout can be acquired [7]. This yields accurate T1 maps but results in long scan times. An efficient T1 mapping has been proposed by Look and Locker [8] where after a single preparation RF-Pulse data is continuously acquired along the recovery curve using a long train of RF-pulses with small flip angles. Due to the application of RF-pulses for the data acquisition, the magnetization recovery changes and multiparametric fitting has been proposed [8, 9], yielding good results for non-moving objects. However, T1-mapping of the abdomen is challenging due to respiratory motion which can lead to ghosting, blurring or signal cancellations in the reconstructed MR images [10] and subsequently errors in the abdominal T1 maps. These errors make it more difficult to detect and to quantify pathologies, possibly causing an inaccurate classification of tissues. The most common approach to minimize respiratory artifacts on T1 maps is a breathhold of the patient [11]. However, this approach requires patient cooperation. Furthermore, breathholding is strenuous, especially for patients, and may not even be feasible for some [11, 12]. This leads to often incomplete breathholds and resulting images that cannot be interpreted. Moreover, breathholding prolongs the overall scan time due to the need of breathing pauses between acquisitions. As the protocol for a diagnostic quantitative imaging sequence is already large, the prolongation of scan time due to breathholding makes it clinically unfeasible to perform a sequence using multiple slice acquisitions for 3D coverage of an abdominal region of interest. For the detection and characterization of multiple liver lesions as well as heterogeneous abdominal pathologies, a 3D coverage of T1 maps is, however, mandatory [13, 14]. 

To overcome this challenge, respiratory gating strategies have been proposed [11], where a respiratory signal is used as a surrogate for the different respiratory motion states of the patient. A wide range of different strategies exits to derive respiratory surrogate signals. Either external devices, such as respiratory bellows [15], optical compression devices [16], the pilot tone [12, 17] as well as a ultrawide band (UWB) radar signal [18], or diaphragmatic 1D navigators [19], self-navigators [20, 21] and image-based self-navigators [22] have been used as motion surrogates. In particular, image-based navigators can be obtained from reconstructed real-time images without the need for any additional hardware or patient setups [23]. They are, therefore, suitable for applications where no external hardware serving as motion surrogate is present, such as in imaging during MR-guided radiotherapy, where T1 mapping can be used for the assessment of liver function in tumors [24–26]. Image-based navigators show promising results for other applications [27–29] as well. However, the techniques were only applied to continuous acquisitions with constant contrast and not for T1 mapping where the contrast changes.

For the correction of respiratory motion and based on any of the aforementioned motion surrogates, data acquisition is only accepted during a predefined respiratory motion state whereas data of any other motion state is either not acquired or rejected for the final image reconstruction. However, as data are only obtained during predefined motion states, scan time efficiency decreases and leads eventually to longer overall scan times for respiratory gating strategies [12]. To overcome these problems, motion-corrected reconstruction strategies have been proposed allowing for data acquisition during free breathing while ensuring high scan efficiencies [23, 30, 31]. For such a motion correction, motion fields are required, which are usually estimated from MR images at different motion states with constant contrast [32, 33]. In T1 mapping, the contrast of the acquired T1-weighted images is changing after magnetization preparation. Motion estimation and motion compensated image reconstruction are, therefore, more difficult for a continuous T1 mapping acquisition. Recently, motion correction has also been applied to improve the efficiency and accuracy of cardiac T1 maps [34].

In this work, we present an approach for respiratory motion-corrected T1 mapping of the abdomen. The presented approach allows the patient to breathe freely during data acquisition. We used an image-based self-navigator, which can handle varying contrasts, as a motion surrogate for the reconstruction of respiratory-resolved images, which were then employed for the estimation of respiratory motion fields. For the assessment of the image-based navigator, a comparison to a simultaneously acquired UWB radar signal was performed. The motion fields were integrated into the T1 mapping process to provide respiratory motion-corrected T1 maps utilizing all the acquired data. The feasibility of the proposed method was evaluated in five healthy volunteers.

## Materials and methods

**Fig. 1:**
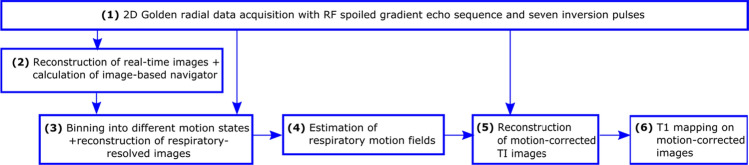
Overview of the proposed motion-correction approach. After data acquisition (**1**), real-time images are reconstructed for the calculation of the image-based navigator (**2**). Using the image-based navigator, the data are binned into different motion states and respiratory-resolved images are reconstructed (**3**). From the respiratory-resolved images, respiratory motion fields are estimated (**4**) and used for the reconstruction of motion-corrected TI images (**5**) to obtain the final motion-corrected T1 map (**6**)

An overview of the proposed approach is given in Fig. 1. The first step (1) is to acquire data using a golden radial acquisition [35] applying multiple magnetization preparation RF-pulses. Such a data acquisition allows for the retrospective reordering of the acquired data to reconstruct images at different time points and using different amounts of data. In the next step (2), real-time images are reconstructed from the acquired data for the calculation of the image-based self-navigator. The image-based self-navigator surrogate is then used to bin the acquired data into different motion states (3) for the reconstruction of respiratory-resolved images. From the respiratory-resolved images, respiratory motion fields (4) are estimated. In the last step, the respiratory motion fields are used to reconstruct motion-corrected images at different inversion times (TI images). From the motion-corrected TI images, motion-corrected T1 maps are calculated (5). In the following, the different steps are described in more detail.

### Data acquisition

Data were acquired at a 3 T MRI scanner (MAGNETOM Verio, Siemens Healthineers, Erlangen, Germany) applying an RF spoiled gradient echo sequence with a flip angle of 5◦, TR = 4.9 ms, TE = 2.2 ms, a resolution of 1.3 mm × 1.3 mm × 8 mm and a field of view (FOV) of 320 mm × 320 mm, with twofold oversampling. For signal reception, a 4-channel flex coil and an 8-channel spine coil from Siemens were used. The total scan duration was 16 s, which is comparable to the time used in the original MOLLI sequence for high-resolution T1 mapping of the heart [36]. During the acquisition, seven equally spaced inversion RF-pulses were applied with the first inversion pulse being executed at 0 s, leading to a time of approximately 2.2 s between inversion RF-pulses. The acquisition of k-space spokes was performed using a 2D golden angle trajectory [35] with 3191 spokes. Coil sensitivities were derived from the same data as used for reconstruction, using all data, independent of the motion states.

### Real-time images and image-based self-navigator

From the acquired k-space data, a sequence of dynamic real-time images was reconstructed using iterative SENSE [37] with five iterations. Each image was reconstructed from 20 k-space spokes, leading to a temporal resolution of 98 ms per image and a total number of 159 images covering the scan time of 16 s. The temporal distance between the real-time images was equal to the temporal resolution, i.e., 98 ms. Due to the application of inversion RF-pulses, the real-time images were acquired at different states of the longitudinal magnetization recovery leading to an image contrast, which changed over time.

**Fig. 2:**
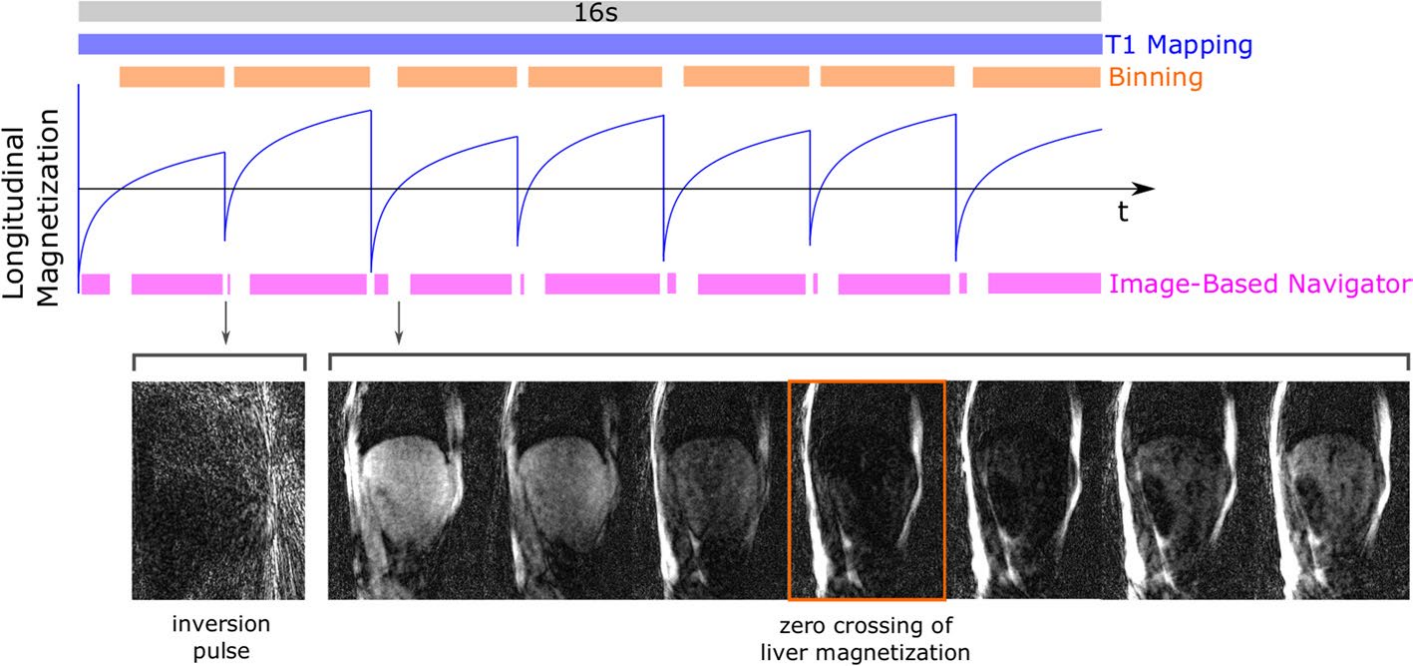
Usage of the data for image-based navigator, binning and T1 mapping. This figure illustrates which parts of the continuously acquired data (grey bar, (**A**)) were used for the calculation of the image-based navigator (magenta bar, (**B**)), for the binning and reconstruction of the respiratory-resolved images (orange bar, (**C**)) and for the T1 mapping (blue bar, (**D**)). Regarding the calculation of the image-based navigator, some of the excluded images are exemplarily shown. The removed images include images acquired during an inversion RF-pulse and images acquired around the zero crossing of the liver magnetization

The reconstructed real-time images were used to calculate an image-based self-navigator. For this, the diaphragm was tracked in a manually drawn region of interest over time. Since the real-time images were reconstructed consecutively in time, the displacement of the diaphragm represents the head-feet respiratory motion of the volunteer in a 2D slice. The position of the diaphragm was given by the corresponding pixel index. With the knowledge of the FOV, the pixel index could be converted into mm, therefore, providing a quantitative motion surrogate. To obtain the final image-based respiratory self-navigator, we interpolated the positions of the diaphragm with a spline-based fit of degree three. The positioning and the size of the region of interest as well as the smoothing factor for the spline interpolation had to be defined manually for each data set. As the position of the diaphragm could not be detected on all reconstructed real-time images due to the application of inversion pulses, a few images were automatically removed for the calculation of the image-based self-navigator. As illustrated in Fig. 2, the automatic removal included images acquired during an inversion pulse and seven images acquired around the zero crossing calculated for healthy liver tissue.

### Respiratory-resolved images

The image-based respiratory navigator signal was used to retrospectively bin the acquired k-space data into ten respiratory motion states. Hereby, each bin consisted of an equal number of spokes (231 spokes, acceleration factor of 3.3 considering the oversampling into readout direction and the factor π/2 for radial sampling) from one motion state, and subsequently, the spacing between respiratory phases varies. A reconstruction of the data from each bin, therefore, leads to respiratory-resolved images with each image representing one of the motion states. In contrast to the real-time images above, each motion state here combines data acquired at various time points after the inversion RF-pulse, resulting in an average contrast that is roughly constant over all reconstructed respiratory-resolved images. To enhance the contrast between lung and liver, we only used k-space data that was acquired for positive values of the longitudinal magnetization of the liver (see Fig. 2). Therefore, data taken 0 to 466 ms after the first inversion and 0 to 338 ms after all other inversions was excluded. These time points were calculated for healthy tissue of the liver. We reconstructed the respiratory-resolved images iteratively using spatial total variation (TV) regularization (12 iterations, spatial variation λ_s_ = 0.0001, same for all volunteers) [38]. No temporal regularization was performed to resolve full end-expiration and end-inspiration.

### Respiratory motion fields

From the respiratory-resolved images, motion fields were estimated using a non-rigid registration algorithm [39]. First, a global affine motion model was estimated, which describes the overall motion of the abdomen. The parameters of the global motion model were then used to initialize the values of the non-rigid registration to estimate the local motion model. The local estimation of motion fields was performed using a free-form deformation (FFD) algorithm based on a cubic B-spline interpolation scheme as deformation model. For the optimal deformation model, an objective function was optimized [40] using normalized mutual information. A spline distance of 10 pixel was used to ensure smooth motion fields and volume preservation was included to obtain physiologically realistic motion fields. For the estimation of motion fields, we chose the first motion state, the end-expiratory state, as a reference state. Each motion field was first estimated relative to the previous motion state and in the end concatenated to all previous motion fields until the first motion state was reached in order to obtain robust motion estimation with low sensitivity to streaking artifacts.

### Motion-corrected TI images and T1 mapping

To carry out motion-corrected T1 mapping, motion-corrected TI images had to be obtained. For this, we first reconstructed TI images using iterative SENSE [37] with 4 iterations. Hereby, all k-space spokes were included (see Fig. 2) with each TI image being reconstructed from 40 k-space spokes, resulting in an acceleration factor of 12.6 per image. Using the image-based self-navigator, each k-space line was assigned to one of the ten motion states. Depending on the assigned motion state of the corresponding k-space spokes, the respective estimated non-rigid motion field obtained from the previous registration step was applied to each reconstructed TI image for non-rigid motion correction in the image space, yielding motion-corrected TI images. Each pixel on the motion-corrected TI images represents the current intensity of the longitudinal magnetization of a certain tissue. To obtain the final motion-corrected T1 maps, we fitted T1, M0 and the flip angle α pixel-wise using the following signal model of the longitudinal magnetization.

### Signal model of longitudinal magnetization

The longitudinal magnetization can be described by the inversion recovery Look-Locker technique [8] with multiple inversions applied at constant time intervals ${T}_{IC}$. The longitudinal magnetization after the j’s inversion is given by [41] ${M}_{z}\left(t\right)={M}_{0}^{eff}-\left({M}_{j}^{+}+{M}_{0}^{eff}\right)\cdot {e}^{-\left(t-\left(j-1\right){T}_{IC}\right)/T{1}^{eff}}$.

After the application of small flip angle pulses, the new steady-state magnetization becomes [41] ${M}_{0}^{eff}=\frac{{M}_{0}T{1}^{eff}}{T1}$ with the effective relaxation time $T1^{eff} = \frac{1}{{1/T1 - \left( {1/T_{R} } \right)\ln \left( {\cos \left( \alpha \right)} \right)}}$ and the repetition time ${T}_{R}$ between the small flip angle pulses.

${M}_{j}^{+}$ describes the longitudinal magnetization right before the j’s inversion. Here, an inversion efficiency of 1 is assumed, leading to the magnetization $-{M}_{j}^{+}$ after the j’s inversion.

### In vivo scans

To study the feasibility of the presented approach, five healthy volunteers (2 female, 26 years and 3 male, 27 years) were scanned in sagittal view. Sagittal images include anterior–posterior as well as head-feet respiratory motion, making it, therefore, possible to obtain motion fields that correct for both main directions of respiratory motion. In order to evaluate the influence of motion correction, scans were carried out during free breathing as well as during a breathhold of the volunteers. For the scans acquired during free breathing, a signal from an external UWB radar system [42] was obtained simultaneously for comparison. All scans were performed in accordance with the institution’s ethical committees, and all the volunteers gave written informed consent before participation.

### Scan time reduction

We evaluated the impact of the scan time on T1 mapping by decreasing the scan time from 16 to 8, 4 and finally 2 s. To obtain the different scan times, the 16 s scan of one exemplary volunteer was retrospectively undersampled to 3191, 1596, 798 and 200 radial spokes. For all different scan times, the motion fields were the same and estimated from a 16 s scan. In this way, the differences in T1 maps were only due to how well T1 rather than how well the respiratory motion could be estimated. This is done as a first outlook on further developments.

### Analysis

To evaluate the quality of the image-based self-navigator, we compared it with the signal obtained from the external UWB radar system [42]. From the UWB radar system, a signal could be extracted that describes the head-feet respiratory motion [16] and which we refer to as the head-feet UWB radar signal. In Ref. [18], it was used to perform respiratory motion-corrected T1 mapping in the liver. Although the head-feet UWB radar signal was not the ground-truth for the respiratory motion, it could serve as a reference for the validation of the image-based self-navigator. However, it was not possible to compare the values quantitatively. This is, because although the image-based self-navigator is a quantitative signal, the head-feet UWB radar signal is only a qualitative one. Therefore, the normalized cross-correlation was used to measure the similarity between both signals. Hereby, the normalized cross-correlation ranged between − 1 and 1 with 1 indicating perfect correlation and − 1 perfect anti-correlation. The head-feet UWB radar signal was recorded with the external hardware and had to be synchronized to the MR data acquisition prior to data analysis.

The reconstructed respiratory-resolved images were evaluated quantitatively with regard to the position of the diaphragm in each image. We further calculated the amplitude of respiratory movement for all volunteers and the amount of respiratory motion that each motion state could resolve.

The motion-corrected T1 maps were qualitatively compared with the uncorrected T1 maps.

Hereby, breathhold T1 maps served as a reference. In particular, a visual assessment of the edge of the liver, the kidney and the blood vessels was carried out with regard to artifact level and feature sharpness.

For the quantitative assessment of the motion-corrected T1 maps, we defined five different regions of interest. The regions of interest included the edge of the liver, the kidney medulla, the kidney cortex, a blood vessel and the region below the blood vessel (see Fig. 6A). For every region of interest, slope graphs were created between the relative difference of the uncorrected T1 to the breathhold values and the relative difference of the motion-corrected T1 to the breathhold values. For each region, the values for all volunteers as well as the average value were shown. We performed statistical tests to evaluate the influence of motion correction on T1 mapping. For normally distributed data, the one-sided student *t*-test for two related samples was used. For data that were not normally distributed, we carried out the one-sided Wilcoxon signed-rank test for two related samples. The statistical test was chosen to be one-sided as motion correction as only a decrease of the relative error to the breathhold values was expected for the motion-corrected T1 in comparison to the uncorrected T1. From the statistical test, the *p*-value could be calculated. For a *p*-value smaller than 0.05, the difference between both groups was considered to be statistically significant. In addition to the slope graphs, the averaged T1 value over all volunteers was analyzed for the motion-corrected and the breathhold case for each region of interest. Statistical tests similar as described before were performed. Last, the standard deviation of T1 over all volunteers was calculated for every region of interest for the uncorrected, motion-corrected as well as the breathhold case.

For evaluating the impact of different scan times on T1 mapping, we examined the T1 maps qualitatively as well as quantitatively. We compared the average T1 values and the corresponding standard deviation for the different scan times for every region of interest. For scan times of 8, 4 and 2 s, the T1 values were compared to the 16 s reference scan. Statistical tests similar as described above were carried out to show if the differences to the 16 s scan could be considered as statistically significant.

## Results

### Image-based self-navigator

The image-based self-navigator could be successfully calculated for all volunteers.

**Fig. 3:**
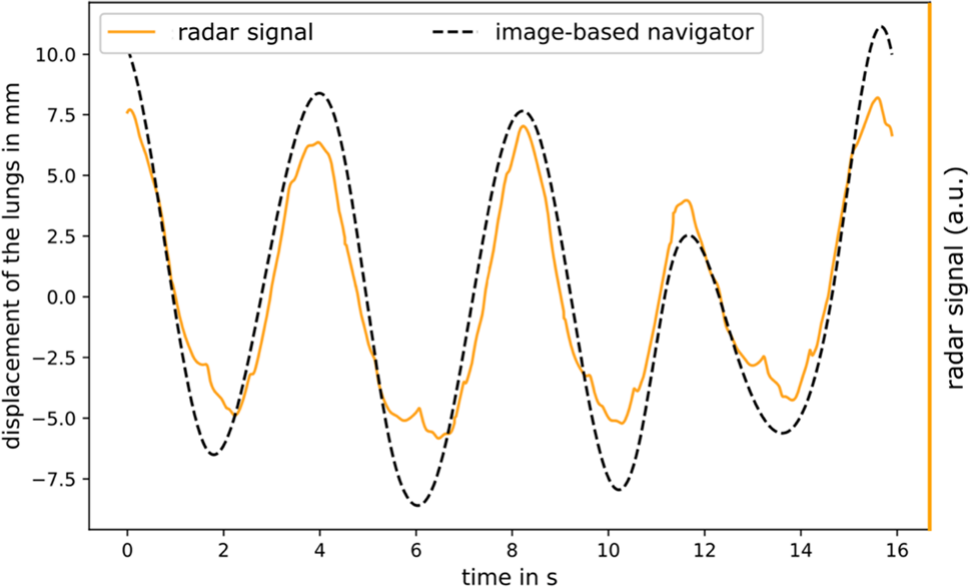
Comparison of image-based self-navigator and head-feet UWB radar signal for one exemplary volunteer. For an easier visual comparison of both signals, the mean value of each signal was removed. Both signals showed a high similarity, with a normalized cross-correlation higher than 0.96 for all volunteers

Figure 3 shows the image-based self-navigator and the corresponding head-feet UWB radar signal exemplarily for one volunteer. Real-time images used for navigator calculation are shown as supplementary material (Online Resource 1). The normalized cross-correlation between image-based self-navigator and head-feet UWB radar signal was higher than 0.96 for all volunteers, illustrating a high similarity between the two signals.

### Respiratory-resolved images

We were able to successfully reconstruct respiratory-resolved images using the image-based self-navigator. Each motion state included on average over all volunteers 2.16 ± 0.5 mm of the respiratory movement, with the same number of spokes in each motion state. A video of the respiratory-resolved images is included as Online Resource 2.

### Respiratory motion fields

**Fig. 4:**
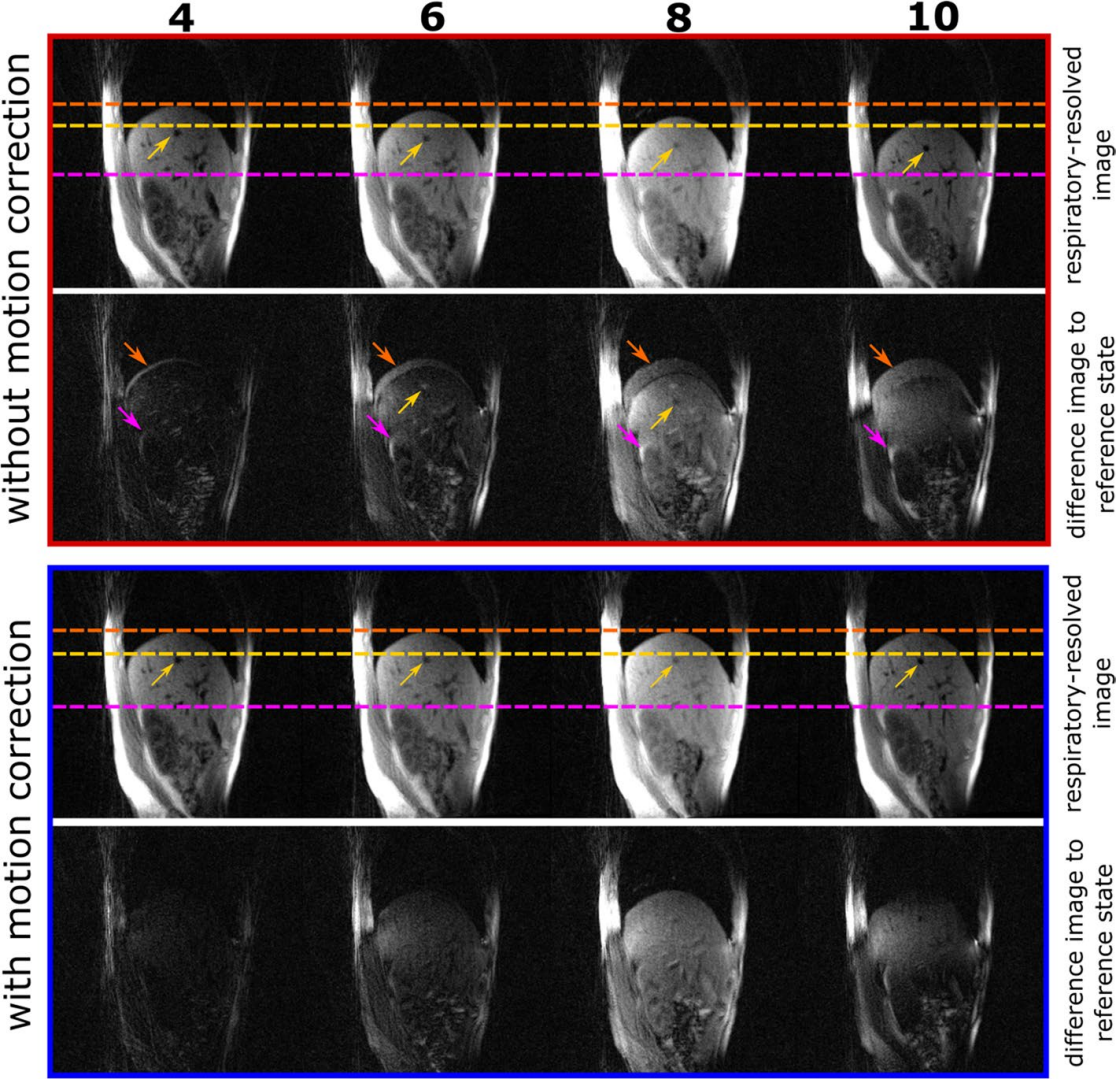
Respiratory-resolved images without and with motion correction. In the first row of the red box, the uncorrected respiratory-resolved images are shown for motion state 4, 6, 8 and 10. In the first row of the blue box, the corrected respiratory-resolved images are shown. The dashed orange line marks the position of the liver edge, the dashed yellow line marks the position of one exemplary blood vessel (indicated by yellow arrows) and the dashed magenta line marks the position of the kidney edge. In the second row of the red box, the difference between the uncorrected images of motion state 4, 6, 8 and 10 and the motion state 1 is shown, respectively. The orange arrows point at the differences at the liver edge, the yellow arrows point at the differences at the blood vessel and the magenta arrows point at the differences at the kidney edge. In the second row of the blue box, the difference between the motion-corrected images of motion state 4, 6, 8 and 10 and the motion state 1 is shown

To evaluate the respiratory motion correction, the estimated motion fields were applied to the respiratory-resolved images to obtain motion-corrected images. A comparison of the uncorrected and these motion-corrected respiratory-resolved images is given in Fig. 4. The uncorrected (first row in red box) and the motion-corrected (first row in blue box) images are shown in motion state 4, 6, 8 and 10. The positions of the liver edge, one blood vessel and the kidney edge in the fourth motion state are marked with dashed horizontal spokes. On the uncorrected images, the positions varied for each motion state due to uncorrected respiratory motion. In contrast, the positions did not change for the different motion states on the motion-corrected images. The successful estimation of motion fields was further shown by the depiction of the difference between the uncorrected images and the image in the first motion state (second row in red box) as well as the difference between the motion-corrected images and the image in the first motion state (second row in blue box). For the uncorrected respiratory-resolved images, differences in image intensity as well as differences at the liver edge and at the kidney edge resulting from the motion of liver and kidney were present. For the motion-corrected respiratory-resolved images, however, only differences in intensity but not in structure were visible. Estimated motion fields are shown in Online Resource 2.

### Motion-corrected T1 maps

Motion-uncorrected as well as motion-corrected TI images are shown in Online Resource 3.

**Fig. 5:**
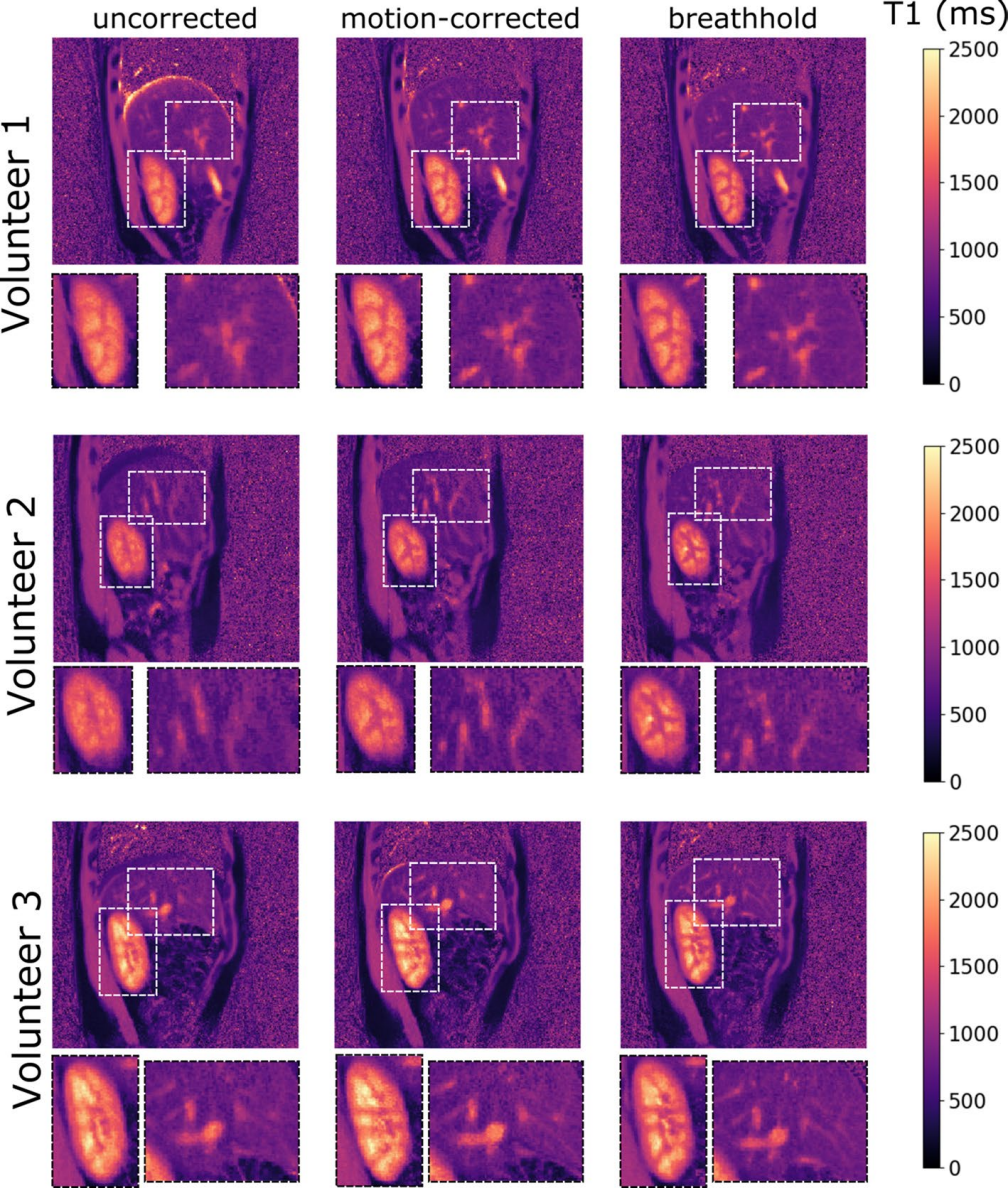
Uncorrected, motion-corrected and breathhold T1 maps for three volunteers and 16 s scan time. Motion-corrected T1 mapping showed less blurring (middle column) compared to uncorrected T1 mapping (left column) for all volunteers. Motion-corrected T1 mapping was visually comparable to breathhold T1 mapping (right column). The respiratory motion amplitude per motion state was 3.09 mm, 1.97 mm and 1.61 mm, respectively, for volunteers 1–3

For all volunteers, the motion-corrected T1 maps qualitatively showed more similarities to the breathhold T1 maps than the uncorrected T1 maps (Fig. 5). At the edge of the liver, the motion-corrected T1 maps exhibit fewer artifacts than the uncorrected T1 maps. The artifacts at the edge of the liver on the uncorrected T1 maps could both result in higher T1 values as well as lower T1 values.

Apart from fewer artifacts at the edge of the liver, the motion-corrected T1 maps further had an impact on the visibility of the blood vessels and the kidney structures (Fig. 5). The kidney cortex was better depicted and the kidney medulla compartments could be distinguished much clearer on the motion-corrected T1 maps. The sharpness of the blood vessels was increased as well.

**Fig. 6:**
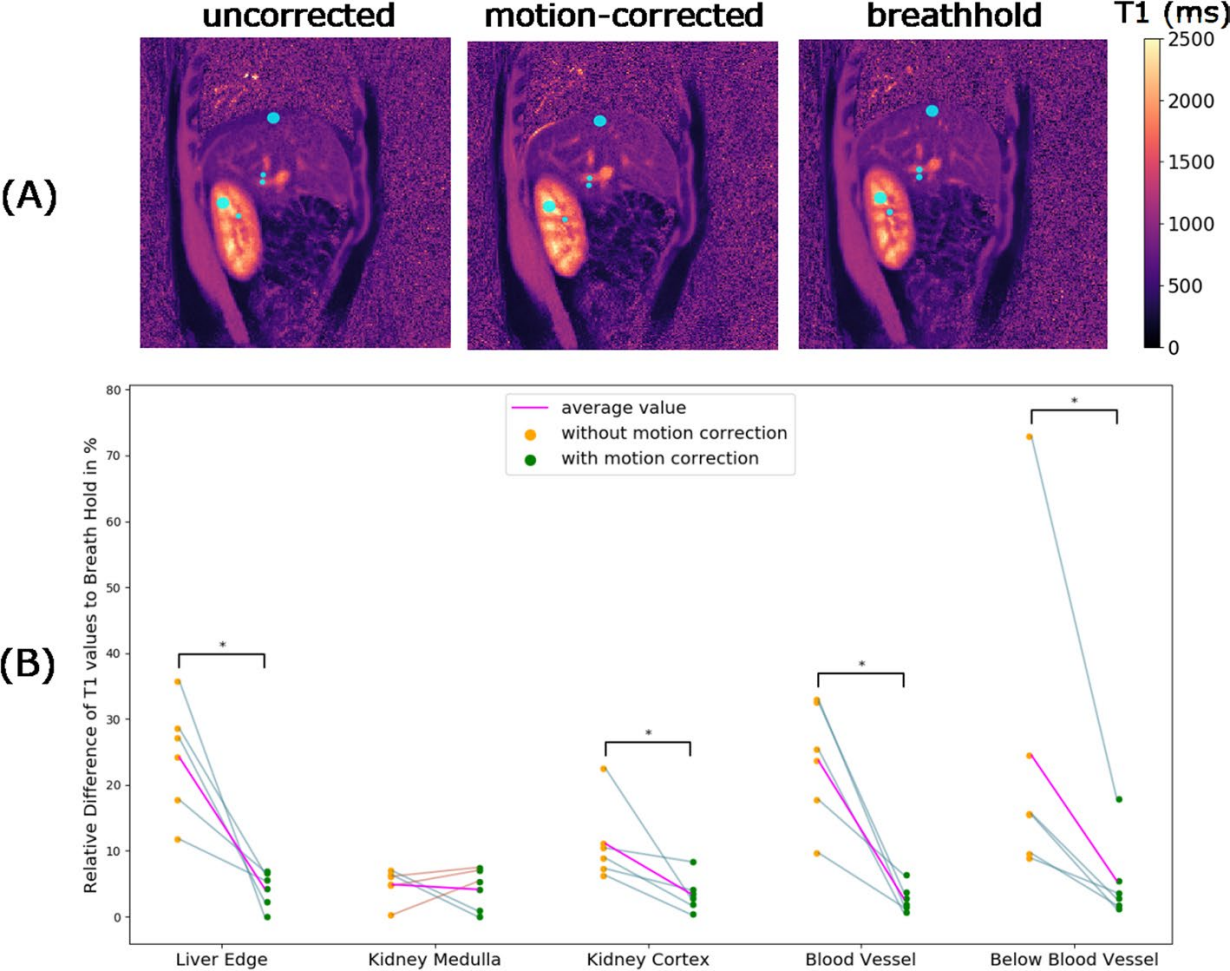
**A** The regions of interest (edge of liver, kidney medulla, kidney cortex, region in and below a blood vessel) that are evaluated quantitatively in (**B**). In **B**, slope graphs of each volunteer are depicted for the chosen regions of interest. The averaged slope graph over all volunteers for each region is shown in magenta, depicting an improvement in T1 mapping for all regions of interest. The significance level between two groups is indicated by brackets. Brackets marked with * indicate significant differences (*p* value smaller than 0.05)

Figure 6B shows the slope graphs for liver edge, kidney medulla, kidney cortex, the blood vessel as well as a region below the blood vessel. We observed that for the regions at the edge of the liver, in the kidney cortex, in and below the blood vessel, the absolute relative difference to the breathhold T1 values was smaller for the motion-corrected T1 values than for the uncorrected T1 values, leading to descending slopes. For these regions, the calculated *p* values further showed that the improvement of T1 values due to motion correction could be considered as statistically significant (*p* < 0.05, indicated by brackets marked with a star sign).

**Fig. 7:**
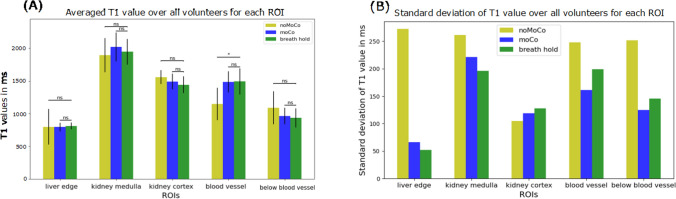
**A** The average T1 values and standard deviation over all volunteers for the uncorrected (noMoCo), the motion-corrected (moCo) and the breathhold case for the regions of interest at the edge of the liver, in the kidney medulla, in the kidney cortex, in as well as below a blood vessel. Brackets marked with ns indicate that no significant differences (*p*-value greater than 0.05) are present, while brackets marked with * indicate significant differences (*p*-value smaller than 0.05). **B** The standard deviation of T1 values over all volunteers for the five regions of interest for the uncorrected, motion-corrected as well as the breathhold case, showing higher variability of T1 values in uncorrected T1 maps compared to corrected T1 maps and breathhold T1 maps

Figure 7A depicts the averaged uncorrected, motion-corrected and breathhold T1 values over all volunteers for each region of interest. The statistical test showed statistically significant differences between uncorrected and breathhold T1 values for the region in the blood vessel. For all other regions, no statistically significant differences between uncorrected and breathhold T1 values could be observed. The statistical test showed no statistically significant differences between motion-corrected and breathhold T1 values for all regions of interest, indicating successful motion correction.

The standard deviations of T1 values over all volunteers for all regions of interest are shown in Fig. 7B. For all regions of interest except the kidney cortex, the standard deviation was highest for uncorrected T1 values, indicating that the range of T1 values over all volunteers was widest for the uncorrected case. After the application of motion correction, the standard deviation decreased.

### Impact of scan time

**Fig. 8:**
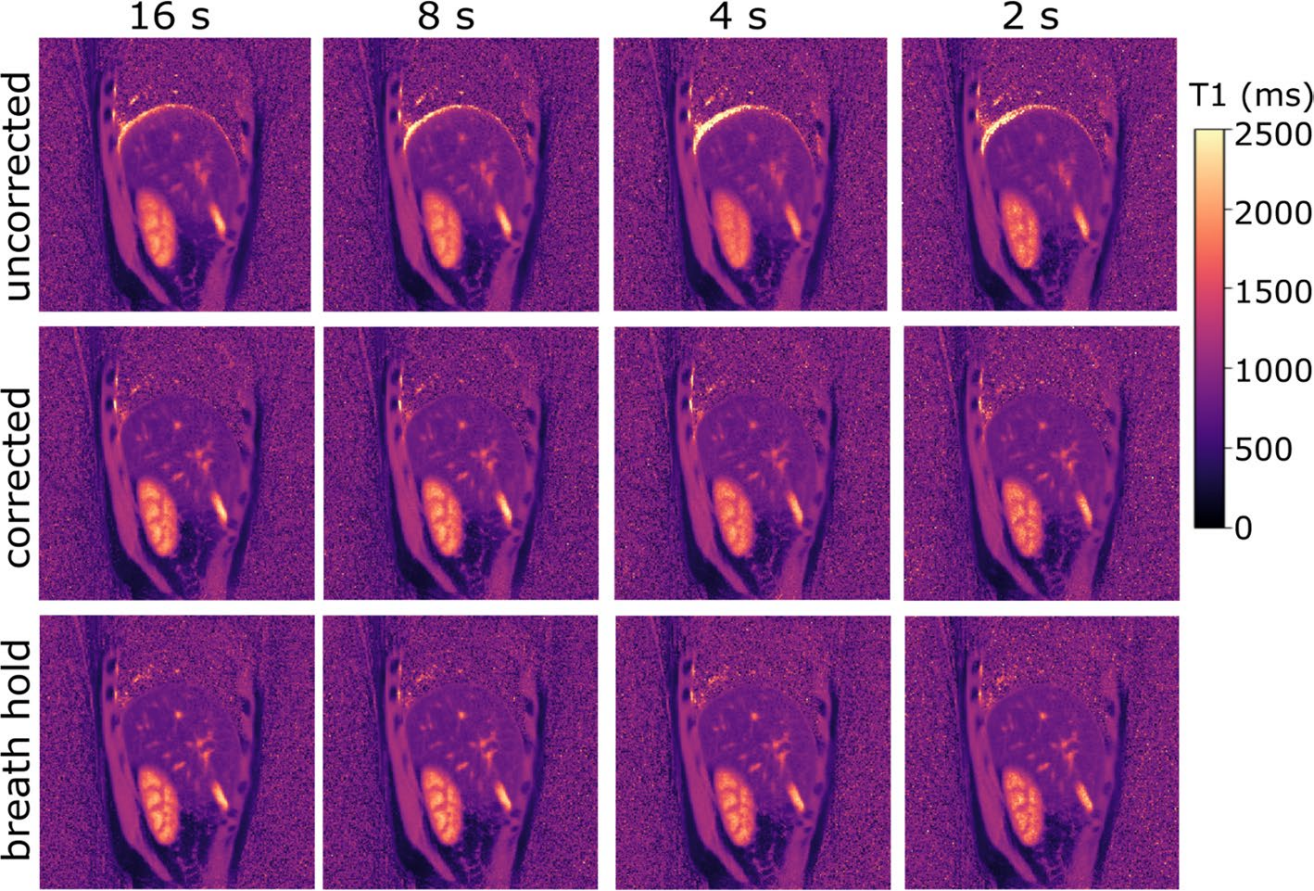
Motion-corrected T1 maps for a scan time of 16, 8, 4 and 2 s. No qualitative differences are visible between the 16 and the 8 s scan as well as between the 16 and the 4 s scan. For a scan time of 2 s, the kidney structures become noisier, which can also be seen in the breath hold image

Figure 8 shows that qualitatively, no differences were visible between the 16 and 8 s scan as well as between the 16 and 4 s scan. For scan durations of 2 s, however, the kidney cortex and the kidney medulla became noisier, making it more difficult to distinguish the kidney structure.

**Fig. 9:**
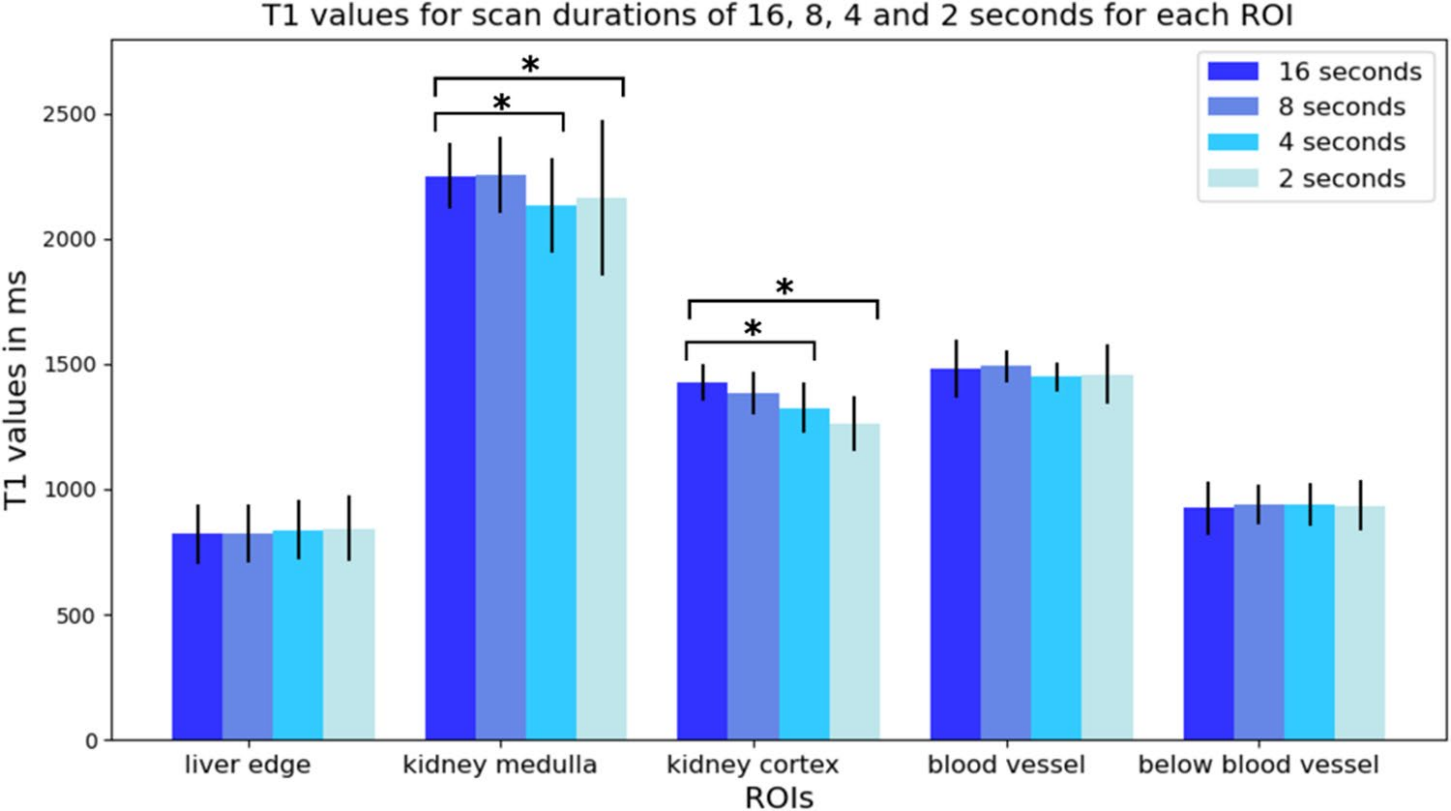
T1 values and standard deviations for different scan times. For each region of interest, the averaged T1 values and corresponding standard deviations over that region are shown for scan durations of 16, 8, 4 and 2 s. Brackets marked with * indicate significant differences (*p* value smaller than 0.05)

In Fig. 9, averaged T1 values and the corresponding standard deviations over the region of interests for scan times of 16, 8, 4 and 2 s are compared. For the liver edge, in and below the blood vessel, there were no statistically significant differences between the values resulting from a 16 s scan and those resulting from scan times of 8, 4 and 2 s. For kidney medulla and kidney cortex, no statistically significant differences between a 16 s scan and an 8 s scan were present. However, between T1 values resulting from 16 s and T1 values resulting from a scan time of 4 and 2 s, we observed statistically significant differences. These images were obtained using the motion field of 16 s to evaluate purely the impact of scan time on the T1 mapping part of the approach. Resulting T1 maps for scan time reduction also including motion estimation are shown in Online Resource 5. Here, reconstruction parameters had to be changed and motion estimation using our approach worked for a scan time of 6 s and longer.

## Discussion

In this study, we presented a method for respiratory motion-corrected T1 mapping of the abdomen. The method uses an image-based self-navigator approach to estimate respiratory motion from the same raw data used for T1 mapping. The motion fields were applied to correct for the respiratory motion yielding motion-corrected T1 maps. The approach was successfully applied in five healthy volunteers. For all volunteers, the motion-corrected T1 maps showed fewer artifacts at the edge of the liver as well as sharper kidney structures and blood vessels. A quantitative evaluation illustrated that the relative error to breathhold T1 maps could be reduced from up to 25% for the uncorrected T1 maps to below 10% for the motion-corrected ones for the average value of a region of interest. For one volunteer, it was possible to reduce the relative error to the breathhold T1 map from 73% for the uncorrected T1 map to 18% for the motion-corrected one. For all assessed regions except the kidney medulla, motion correction lead to a statistically significant improvement of T1 mapping. The improvement for the kidney medulla is not statistically significant as it is in the center of the kidney and, therefore, less affected by motion. A comparison between the averaged motion-corrected and breathhold T1 values over all volunteers showed that there were no statistically significant differences.

The standard deviation of T1 values over all volunteers was lower for the motion-corrected T1 mapping compared to motion-uncorrected T1 mapping. If the range of healthy T1 values is small for healthy volunteers, it is easier to define a pathological window. Thus, pathological tissue could potentially be distinguished with higher certainty from healthy tissue. Therefore, motion correction could help to improve the detection of pathologies in the abdominal region. Especially, in border regions, for example between liver and lung, motion artifacts lead to uncertain T1 values, limiting the interpretability of the maps.

In general, the results from the performed motion-corrected T1 mapping in the abdomen were not compared to any phantom or independent reference measurements. Phantom measurements were not carried out, because to the best of our knowledge, there is no phantom which yields realistic non-rigid respiratory motion and realistic T1 values. No independent reference measurements were carried out. Nevertheless, in Ref. [30], comparable T1 values for the liver were obtained (781 ± 90 ms in Ref. [43], 795 ± 67 ms in Fig. 7A for the liver edge on motion-corrected T1 maps). In our feasibility study, only five volunteers were included. For further clinical use, more volunteers and patients should be added.

While self-navigation was already used in dynamic imaging of constant contrast including additional self-navigation spokesin the acquisition[27, 32, 44], we showed that it could also be applied for quantitative imaging with varying contrasts using the same data as used for T1 mapping for a high T1 mapping scan efficiency. For the calculation of the image-based self-navigator, the position and the size of the region of interest as well as the smoothing factor had to be defined manually for each volunteer. In the future, an automated optimization/segmentation could be implemented. An interpolation of the diaphragm positions for the calculation of the image-based self-navigator might not be necessary with a higher temporal resolution of the real-time images. For achieving high temporal resolution, sliding window (SW) reconstruction [45] could be a possible approach. However, reconstruction time can be extremely long for SW technique if dedicated reconstruction hardware or high-performance workstations are not available [46]. For our image-based self-navigation approach, seven images were excluded during zero-crossing of the signal to ensure high signal intensities of the liver for a wider range of heathy and pathological T1 times of the tissue to obtain a robust navigator. This could be reduced in the future by patient-wise exclusion of images, depending on, e.g., the noise level of the images to obtain a higher temporal resolution around zero-crossings.

The calculated image-based self-navigator showed very good correlation with the acquisition-independent head-feet UWB radar signal obtained from the external UWB radar. Therefore, the image-based self-navigator is suitable as a motion surrogate in T1 mapping without the need for external hardware. Moreover, in comparison to the head-feet UWB radar signal, the image-based self-navigator was able to provide quantitative information about the respiratory motion instead of only qualitative one.

The respiratory-resolved images could be successfully reconstructed, giving an average resolution of 2.16 ± 0.5 mm for each motion state. The relatively high value was mostly caused by one single volunteer, who had a much higher breathing amplitude than the other volunteers. However, the volunteer with smallest respiratory motion still had an average resolution of 1.61 mm, which is larger than the voxel-size. Instead of defining the same number of motion states for all volunteers, the number could be determined individually for each volunteer in the future. Each motion state should have a resolution, which is smaller than the spatial resolution of the reconstructed images in order to ensure that all motion can be corrected for.

The motion fields estimated using a non-rigid registration algorithm were sufficient to successfully correct for motion in our images. However, to further improve image registration, advanced strategies such as groupwise image registration [47] or deep-learning-based registration methods [48] could be employed.

The evaluation of the scan time showed that the scan times could be decreased to 8 s, as the 8 s scan showed no qualitative as well as no statistically significant differences to the 16 s scan. However, the scan time was only reduced for the reconstruction of the TI images, while the motion fields were still estimated from the complete 16 s scan. Therefore, it was only possible to perform a part of the approach with decreased scan time. Figure 8, however, shows that motion correction is also crucial for shorter scan times. To be able to actually reduce the scan time for the whole approach, the reconstruction of respiratory-resolved images and the estimation of motion fields should thus also be made possible with a decreased scan time. However, this is challenging due to differences in contrast of the motion-resolved images. To overcome this challenge, different regularization factors for image reconstruction and motion estimation have to be found (Online Resource 4). With a decreased scan time of 8 s, a short breathhold could be considered instead of the presented motion-correction approach. However, in case of a 3D scan, longer scan times are needed. An extension of our 2D approach to 3D would be, therefore, desirable in the future to correct for motion in 3D scans.

Although an overall improvement could be achieved with the motion-corrected T1 maps, the visual quality of the breathhold T1 maps was still slightly better compared to the motion-corrected T1 maps. However, it has to be taken into account that also the breathhold T1 maps could potentially suffer from incomplete breath holding. In addition, differences in the T1 maps could be further caused by differences in expiration motion states between the breathhold and the motion-corrected T1 map acquisitions. The quantitative T1 values on the motion-corrected T1 maps also did not exactly match the values on the breathhold T1 maps. This is most likely due to the fact that the T1 maps were acquired during different scans, making it, therefore, not possible to choose exactly the same region of interest on the two T1 maps for comparison. Another potential reason is through-plane motion for which a correction could not be carried out in our 2D approach.

The image quality of T1 maps could be further enhanced by improving motion estimation and by increasing the quality of the TI images and T1 maps using other advanced reconstruction techniques, such as compressed sensing or model-based reconstruction methods [41, 49–51]. For low-rank motion-corrected reconstruction for quantitative imaging, the image-based navigator could be equally used instead of an external bellow [52]. The mentioned reconstruction techniques could be integrated in our approach in the future. In this study, the proposed approach was applied to conventional T1 mapping. However, respiratory motion is also present in abdominal MR fingerprinting, with the advantage of simultaneous acquisition of multiple quantitative parameter [53] and liver MR elastography [54]. The presented motion-correction approach could be considered for these techniques as well instead of breathholding [55, 56].

In conclusion, an approach for respiratory motion-corrected T1 mapping of the abdomen was presented which allowed for free-breathing T1 mapping in a short scan time. Using an image-based self-navigator, no external respiratory signal was needed. Motion correction reduced the motion-induced T1 mapping error from 25 to 10% for the average value of a region of interest, and from 73 to 18% for one volunteer. The total scan time could possibly be further decreased to 6–8 s.

## Supplementary Information

Below is the link to the electronic supplementary material.

Supplementary file1 (DOCX 13 KB)

Supplementary file2 (AVI 168309 KB)

Supplementary file3 (MP4 10248 KB)

Supplementary file4 (AVI 168309 KB)

Supplementary file5 (PNG 4031 KB)

Supplementary file6 (PNG 106 KB)

Supplementary file7 (PNG 75 KB)

Supplementary file8 (PNG 74 KB)

Supplementary file9 (PNG 108 KB)

Supplementary file10 (PNG 125 KB)

## Data Availability

Raw data were generated at Physikalisch-Technische Bundesanstalt Berlin. Derived data supporting the finding of this study are available from the corresponding author Jana Huiyue Zhang on request.
