# Supplementary material for: Respiratory motion-corrected T1 mapping of the abdomen
Source: MAGMA. 2024 Aug 12;37(4):637–49. doi: 10.1007/s10334-024-01196-1 (PMC11417068; doi:10.1007/s10334-024-01196-1)
Supplement: Supplementary file 1 — Supplementary file1 (DOCX 13 KB) [file 10334_2024_1196_MOESM1_ESM.docx]

**Captions for supplementary material**

**Online Resource 1**

Online resource 1 shows the real-time images.

**Online Resource 2**

Online resource 2 shows the respiratory-resolved images without motion correction (left), the estimated motion fields of the respiratory-resolved images (middle) and the motion-corrected respiratory-resolved images (right).

**Online Resource 3**

Online Resource 3 shows the motion-uncorrected as well as the motion-corrected TI images.

**Online Resource 4**

Online Resource 4 shows different regularization factors for image reconstruction and motion estimation for scan times ranging from 16 s to 3 s. The first row shows the uncorrected T1 maps, the second row the T1 maps that were reconstructed and motion-corrected for with the standard regularization factors. The third row shows the motion-corrected T1 maps using adapted regularization factors.

**Online Resource 5**

Online Resource 5 shows the calculated image-based navigator and the UWB radar signal for a volunteer.

**Online Resource 6**

Online Resource 6 shows the calculated image-based navigator and the UWB radar signal for a volunteer.

**Online Resource 7**

Online Resource 7 shows the calculated image-based navigator and the UWB radar signal for a volunteer.

**Online Resource 8**

Online Resource 8 shows the calculated image-based navigator and the UWB radar signal for a volunteer.

**Online Resource 9**

Online Resource 9 shows the calculated image-based navigator and the UWB radar signal for a volunteer.

**Online Resource 10**

Online Resource 10 shows the uncorrected, motion-corrected and breathhold T1 maps for an obese volunteer (BMI of 31,2 kg/m²) as well as the corresponding image-based navigator.
