# Supplementary figures and images for: Respiratory motion-corrected T1 mapping of the abdomen
Source: MAGMA. 2024 Aug 12;37(4):637–49. doi: 10.1007/s10334-024-01196-1 (PMC11417068; doi:10.1007/s10334-024-01196-1)

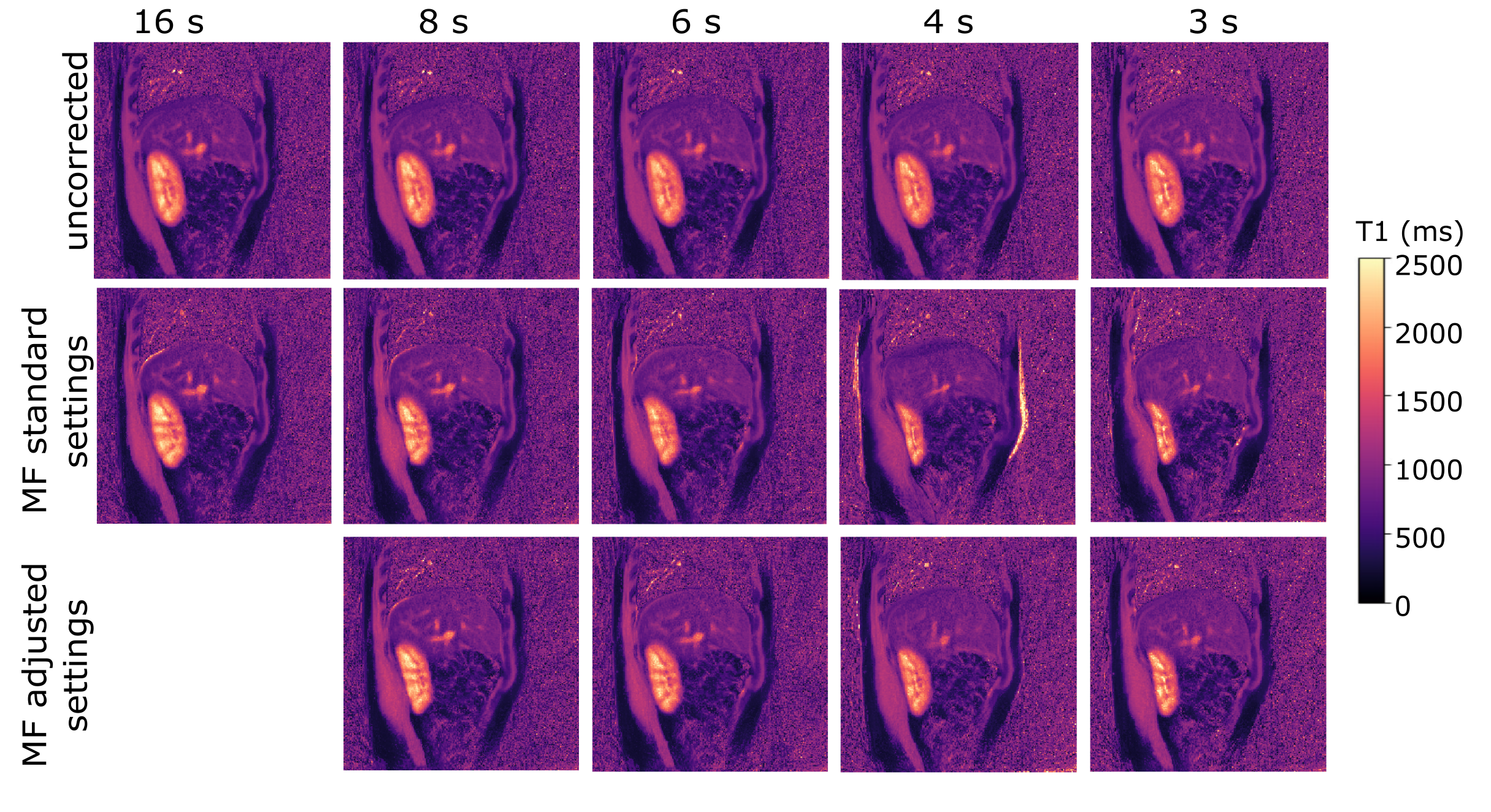

Supplement: Supplementary file 5 — Supplementary file5 (PNG 4031 KB) [file 10334_2024_1196_MOESM5_ESM.png]

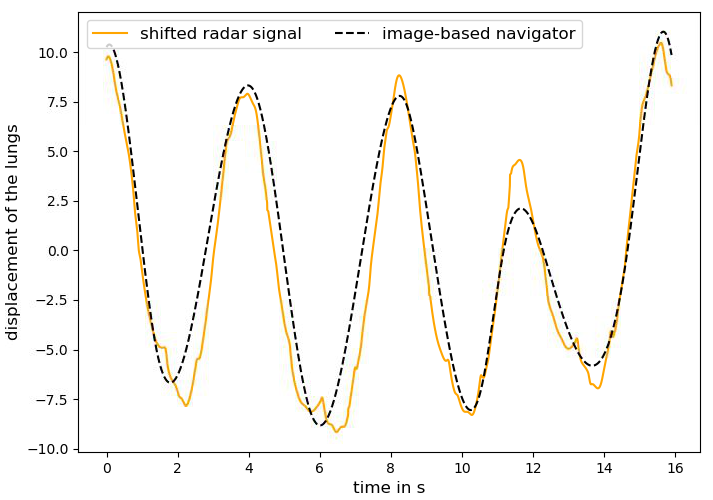

Supplement: Supplementary file 6 — Supplementary file6 (PNG 106 KB) [file 10334_2024_1196_MOESM6_ESM.png]

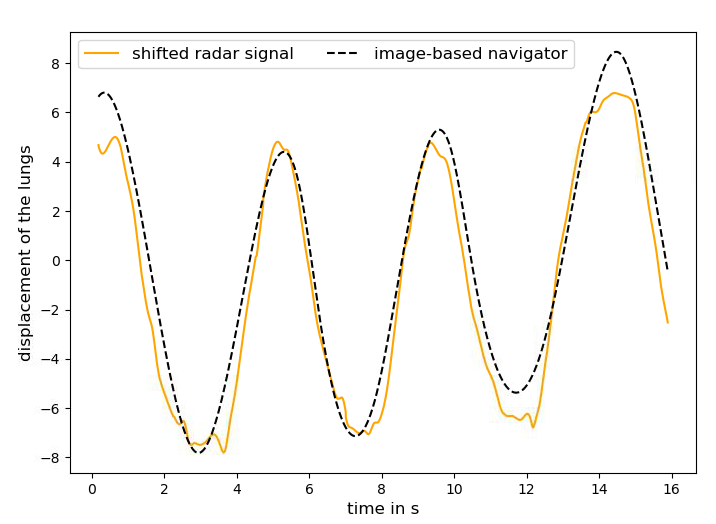

Supplement: Supplementary file 7 — Supplementary file7 (PNG 75 KB) [file 10334_2024_1196_MOESM7_ESM.png]

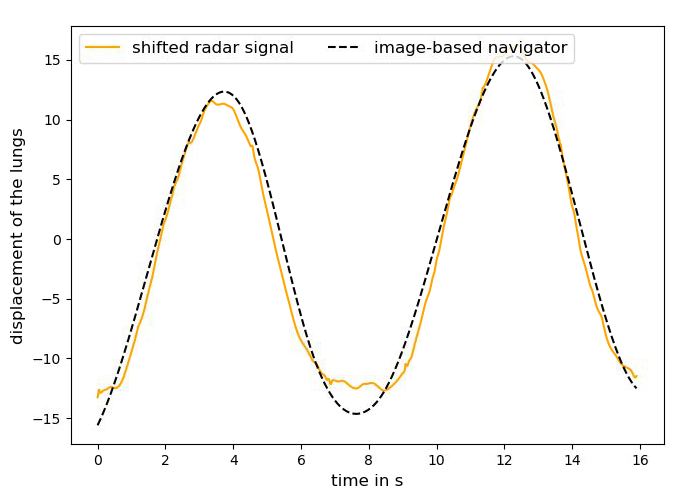

Supplement: Supplementary file 8 — Supplementary file8 (PNG 74 KB) [file 10334_2024_1196_MOESM8_ESM.png]

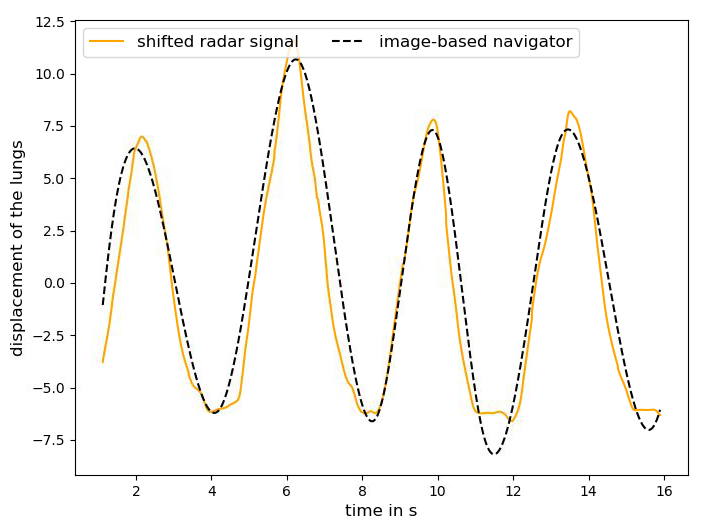

Supplement: Supplementary file 9 — Supplementary file9 (PNG 108 KB) [file 10334_2024_1196_MOESM9_ESM.png]

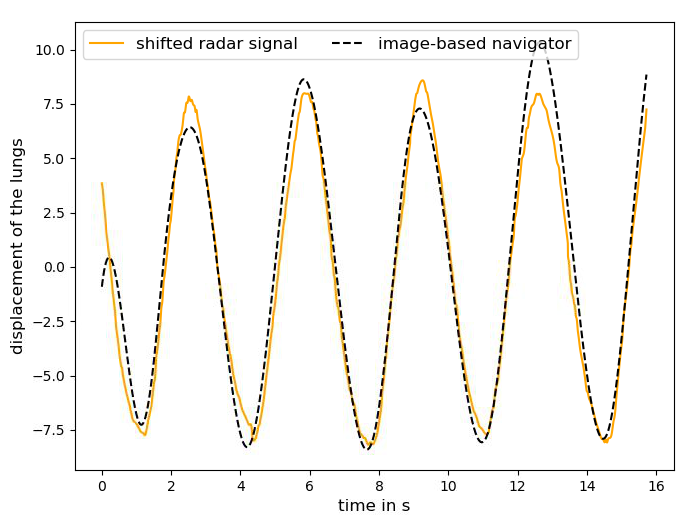

Supplement: Supplementary file 10 — Supplementary file10 (PNG 125 KB) [file 10334_2024_1196_MOESM10_ESM.png]
